# Has learning gone to waste?—Health-promoting behaviors of seniors

**DOI:** 10.3389/fpubh.2024.1403496

**Published:** 2024-07-05

**Authors:** Józefa Dąbek, Magdalena Szynal, Oskar Sierka, Ewelina Łebek, Halina Kulik

**Affiliations:** ^1^Department of Cardiology, Faculty of Health Sciences in Katowice, Medical University of Silesia in Katowice, Katowice, Poland; ^2^Doctoral School of the Medical University of Silesia in Katowice, Medical University of Silesia in Katowice, Katowice, Poland; ^3^Department of Propaedeutics of Nursing, Faculty of Health Sciences in Katowice, Medical University of Silesia in Katowice, Katowice, Poland

**Keywords:** seniors, healthy behavior, Universities of Third Age, education, aging

## Abstract

**Objective:**

Regardless of the fact that Universities of Third Age (UTA's) are becoming more and more popular among seniors there are not many available studies examining the impact of conducted educational activities on seniors' adherence to health-promoting activities. The aim of the study was to compare health behaviors (e.g.,: physical activity, eating habits, alcohol consumption, tobacco smoking, preventive tests performance) between seniors attending and not attending UTA's classes.

**Methods:**

The study involved 631 (100%) seniors aged 60–92 years (x =70.28 ± 6.09 years). The majority of the study group were women (475; 75.28%). To conduct the study, a proprietary questionnaire was used, consisting of questions regarding the discussed topic and basic questions including: age, gender, place of residence and education. Polish versions of standardized questionnaire—“*My eating behaviors”* examine eating behaviors of the respondents. The Chi^2^ test was used for qualitative data, and for quantitative data—the Mann-Whitney U test (No normal distribution: T S-W < 0.001). Linear and logistic regression models were used to check whether the associations would remain after adjustments for potential cofounders. The level of statistical significance was set at α < 0.05.

**Results:**

Number of seniors participating in UTA's activities was higher in terms of engaging in: actively spending free time (261; 73.73% vs. 93; 26.27%; *p* < 0.001), regular physical activity (270; 76.27% vs. 133; 48.01%; *p* < 0.001), self-assessment of physical activity (259; 73.16% vs. 95; 26.84%; *p* = 0.004), duration of physical activity (< 0.001), past tobacco smoking (133; 37.57 vs. 76; 27.44%; *p* = 0.007) and alcohol consumption depending on the habit frequency (*p* < 0.001). Number of seniors not participating in UTA's classes was lower in terms of: regular annual dental controls (161; 58.12%; vs. 265; 74.86%; *p* < 0.001), regular self-examination of breasts/testicles (148; 53.43% vs. 218; 61.58%; *p* = 0.04) and regular laboratory tests (232; 83.75% vs. 318; 89.83%; *p* = 0.02).

**Conclusions:**

Health-promoting behaviors of seniors attending classes at the UTA's were more correct in terms of physical activity, adequate attendance with preventive test and worst in terms of alcohol consumption. Overall picture allows to conclude that participation in UTA's classes seems to have a positive impact on the examined health-promoting behaviors of the surveyed seniors.

## Introduction

The World Health Organization (WHO) has defined health as “a state of complete physical, mental and social well-being and not merely the absence of disease or infirmity” (1, 2).

According to Lalonde's concept of “health fields”, it can be clearly seen that lifestyle has the greatest impact on human health and constitutes 50% of the factors determining it. Other factors are: the environment (all external elements surrounding a person)-−20%, the biology of the human body (sex, age, genetic factors)-−20% and the organization of medical care−10% (3–6).

In every age group, including seniors, prevention is particularly important in order to eliminate harmful risk factors for many diseases, including the circulatory system and cancer (7). The diseases mentioned are the most common diseases in the world. Elements of an unhealthy lifestyle including: improper diet, overweight/obesity, smoking, little or no physical activity and alcohol abuse strongly contribute to their development (8).

The above-mentioned information has become the basis for the development of health promotion worldwide, and the concept of “healthy lifestyle”—conscious action aimed at improving or maintaining health—has gained popularity (3). A healthy lifestyle includes, among others: diet, physical activity, not using stimulants (not smoking, limiting alcohol consumption) and monitoring one's own health by e.g., performing regular preventive test.

According to available studies adherence to health behaviors among seniors is not good enough and the importance and need of behavioral interventions in this area were highlighted in the literature (9, 10). Liu et al. in their study regarding adherence to health promoting behaviors of seniors ≥60 years of age stated that full adherence rate to blood pressure monitoring among 19,800 participants was lover than 20% (11). Agrawal S. et al. showed in their study of numerous health-promoting activities that out of 1,000 adult Poles examined, the percentage of people performing blood lipid tests was approximately 59.1%, and controlling glucose concentration 65.8% (12). Whereas, Calas et al. found that among 2,620 participants of their study, only about 49% engaged in physical activity and Zaragoza-Marti et al. in the group of 341 respondents older than 60 years, found that many of them has nutritional deficits e.g., in in the case of vitamin D, essential for maintaining good bone health, and iodine, important for endocrine–metabolic control (13, 14).

In Poland, the founder of the first University of the Third Age (UTA's) established in 1975 in Warsaw was prof. Halina Szwarc. It was a part of the Postgraduate Center for Medical Staff Education. Its assumptions were: the possibility of educating seniors who could not receive education in their youth and improving the quality of their lives, as well as implementing a continuing education program and conducting gerontological research.

Thanks to participation in UTA's classes, seniors can develop and pursue their youthful interests and passions through various forms of self-education, including: getting to know the environment, being in a group, acceptance, expanding knowledge and skills, filling free time, learning new technologies and ways of communicating. UTA's also help meet the psychosocial and health needs of seniors (15). In addition, they counteract the marginalization of seniors, and participation in the offered activities ensures good wellbeing, stress reduction and increased physical activity (16, 17). Many UTA's offer classes on health education and learning healthy habits, which are very popular among seniors. UTA's also organizes many meetings with experts and other events on health prevention, from which participants can gain knowledge about a healthy lifestyle (18).

Regardless of the fact that UTA's is becoming more and more popular among seniors there are not many available studies examining the impact of educational activities on seniors' adherence to health-promoting activities.

The aim of the study was to compare health behaviors (e.g.,: physical activity, eating habits, alcohol consumption, tobacco smoking, preventive tests performance) between seniors attending and not attending UTA's classes.

## Materials and methods

The cross-sectional study was conducted after obtaining consent from the Bioethics Committee of the Medical University of Silesia in Katowice (PCN/0022/KB1/36/21). All methods used in this study were consistent with applicable guidelines and regulations on conducting scientific research, and all seniors gave their informed consent to participate in it.

The participants inclusion criteria for the study included: voluntary, informed consent to participate in the study, ability to follow instructions, ability to read and no need for help from other people in completing the questionnaire.

The study involved 631 (100%) seniors from Silesia Voivodeship, Poland. Participants were recruited from Polish Universities of Third Age attendees (354 seniors) and among researcher's families' members and friends, as well as the researchers' neighbors (277 seniors) who did not participate in Universities of Third Age classes/lectures. The above-mentioned respondents who did not attend UTA's classes were also asked to give the study sets, described below, to their friends and families' members. It can therefore be concluded that the snowball method was used to recruit the seniors not attending UTA's classes. Data were collected from 2019 to 2020.

To conduct the study, an original survey questionnaire was used, consisting of questions regarding the discussed topic and basic questions including: age, gender, place of residence and level of education. Questions assessing health-promoting behaviors in the field of: how to spend free time, regularity of physical activity, self-assessment of physical activity, data on smoking, alcohol consumption and regularity of diagnostic tests and visits to the doctor were the proprietary questions designed by authors. Eating behaviors were assessed using a Polish standardized questionnaire—“*My Eating Behaviors”* (19). The internal consistency of the questionnaire was examined using the Cronbach's alpha coefficient—alfa = 0.82.

Respondent form UTA's “+” group were invited to participate in the study before and during breaks in UTA's classes. Each respondent, who gave informed consent to participate, received the survey questionnaire in an unmarked white envelope (study set) and sat at a prepared table at a distance that made it impossible to see the responses of another survey participant or communicate. After completing the survey, the respondent placed the paper questionnaire in the above-mentioned white unmarked envelope, which was sealed and placed in a prepared closed box. After filling the box with envelopes, the researchers transported them to the science lab, where the box was opened. Then, the researchers opened individual envelopes, took out the completed questionnaires and entered the selected answers into the database. A similar procedure applied to UTA“–” participants. After obtaining their informed consent to participate in the study, they received the study set and completed the questionnaires independently in their homes. They also passed mentioned sets to their family members and friends. Sealed envelopes with answers and signed informed consents were handed over to the researchers and transported to the science lab. The further procedure was the same as for UTA”+” participants. As mentioned before completing the study questionnaire was completely anonymous and voluntary. The methods used to collect the questionnaires (placing them in white unmarked envelopes after completing them, collecting the envelopes in one secured place, opening them only when entering the obtained results into the database) made it impossible to identify the participants of the study.

For the purposes of the analyses, the surveyed seniors were divided depending on their attendance at classes at UTA's. Statistica 13.3 (StatSoft Poland) was used to perform statistical analyses. The Chi^2^ test was used for qualitative data, and for quantitative data—the Mann-Whitney U test (No normal distribution: T S-W < 0.001). Further analyses using multiple and logistic regressions models were used to identify whether the associations would remain after adjustments for potential cofounders (categorical: sex, place of residence, UTA's participation and continuous: age). There was no missing data. The level of statistical significance was set at α < 0.05.

## Results

Table 1 shows the general characteristics of the study group.

**Table 1:** General characteristics of the study group (*n* = 631).

**Variables**	**Data**	
	***n***	**%**
**Sex**		
Male	156	24.72
Female	475	75.28
**Age [years]**		
60–70	373	59.11
71–80	220	34.87
≥81	39	6.18
**Level of education**		
Primary	37	5.86
Vocational	120	19.02
Secondary	292	46.28
Higher	182	28.55
**Place of residence**		
City	436	69.10
Village	195	31.90
**Attending classes at Universities of the Third Age**		
Yes	354	56.10
No	277	43.90
**Marital status**		
Single	26	4.12
Married	355	56.26
Widow/widower	224	35.50
Informal relationship	26	4.12
**Residence**		
Alone	206	32.65
With spouse	306	48.49
With family	109	17.27
In a nursing home	2	0.32
With a partner	8	1.27
**Material situation**		
Good	164	25.99
Average	313	49.60
Below average	126	19.97
Bad	23	3.65
Neither good, nor bad	5	0.79

Data are presented as absolute frequencies.

Participants were from 60 to 92 years of age ($\bar{\text{x}}$ =70.28 ± 6.09 years). The majority of the surveyed group were women (475; 75.28%), and most respondents had secondary education (292; 46.28%). Approximately 32% (195; 31.90%) of the surveyed seniors lived in rural areas, and over 55% (354; 65.10%) of the respondents declared that they participated in classes at UTA's.

Table 2 presents the characteristics of the study group of seniors, including physical activity according to the participation in the UTA's.

**Table 2:** Physical activity according to the participation in the University of the Third Age (*n* = 631).

**Variables**	**UTA “**+**” (*****n*** = **354)**		**UTA “–” (*****n*** = **277)**		***P*-value**
	***n***	**%**	***n***	**%**	
**Spending free time**					
Active (e.g., walking, swimming)	261	73.73	125	45.13	< 0.001
Passively (e.g., armchair/sofa)	93	26.27	152	54.87	
**Regular physical activity**					
Yes	270	76.27	133	48.01	< 0.001
No	84	23.73	144	51.99	
**Self-assessment of physical activity**					
I am a physically active senior	259	73.16	173	62.45	0.004
I am not a physically active senior	95	26.84	104	37.55	

Data are presented as absolute frequencies, UTA “+”—seniors participating in classes at Universities of the Third Age, UTA “–”—seniors refusing to attend classes at Universities of the Third Age, P-value—statistical significance.

The proportion of seniors more often declaring spending their free time actively was higher among those attending UTA's classes than those who did not (261; 73.73% vs. 125; 45.13%). The Chi^2^ test confirmed the statistical significance of the observed differences (p < 0.001). The above-mentioned test also confirmed that number of seniors participating in UTA's activities was higher in terms of engaging in regular physical activity (270; 76.27% vs. 133; 48.01%; *p* < 0.001) and describing themselves as physically active (259; 73.16% vs. 173; 62.45 %; *p* = 0.004).

Table 3 presents the characteristics of the studied group of seniors, including descriptive statistics and the results of the differences analysis depending on the time of physical activity undertaken, numbers of point obtained in the “My Eating Habits” questionnaire and participation in activities of UTA's.

**Table 3:** Descriptive statistics and the results of the differences analyses depending on the time of physical activity undertaken, numbers of point obtained in the “My Eating Habits” questionnaire and participation in activities of Universities of the Third Age.

**Variables**	**UTA “+” (*n* = 354)**	**UTA “–” (*n* = 277)**	**The entire study group**
**Time to engage in physical activity [minutes]**			
Median	30	0	25
Lower quartile	10	0	0
Upper quartile	60	30	60
**Number of points obtained in “My Eating**			
**Habits” questionnaire**			
Median	10	10	10
Lower quartile	7	7	7
Upper quartile	13	12	13
**Analysis of differences in the:**			***P*** **-value**
Duration of physical activity			< 0.001
Number of points obtained in the “My Eating			0.23
Habits” questionnaire			

Data are presented as absolute and relative frequencies or median and interquartile range. UTA “+”—seniors participating in classes at Universities of the Third Age, UTA “–”—seniors refusing to attend classes at Universities of the Third Age, p-value—statistical significance.

The Mann-Whitney U test showed that the analyzed groups of seniors differed significantly in terms of time spent on physical activity during the day (*p* < 0.001), but not in terms of the points obtained in the “My Eating Habits” questionnaire (*p* = 0.23).

Table 4 presents the characteristics of the study group of seniors, including alcohol and tobacco smoking habits according to the participation in the in the UTA's.

**Table 4:** Alcohol and tobacco smoking habits according to the participation in the in the University of the Third Age (*n* = 631).

**Variables**	**UTA “**+**” (*****n*** = **354)**		**UTA “–” (*****n*** = **277)**		***P*-value**
	***n***	**%**	***n***	**%**	
**Currently tobacco smoking**					
Yes	30	8.47	27	9.75	0.580
No	324	91.53	250	90.25	
**Tobacco Smoking in the past**					
Yes	133	37.57	76	27.44	0.007
No	221	62.43	201	72.56	
**Passive tobacco smoking**					
Yes	54	15.25	56	20.21	0.103
No	300	84.75	221	79.78	
**Alcohol consumption**					
At all	121	34.18	131	47.29	< 0.001
Every day	8	2.26	21	7.58	
2–3x a week	32	9.04	19	6.86	
Once a month	88	24.86	44	15.88	
Several times a month	27	7.63	19	6.86	
Several times a year	78	22.03	43	15.52	

Data are presented as absolute and relative frequencies or median and interquartile range, UTA “+”—seniors participating in classes at Universities of the Third Age, UTA “–”—seniors refusing to attend classes at Universities of the Third Age, p-value—statistical significance.

Tobacco smoking was declared by only 57 (9.03%) seniors in total, and the difference in the number of respondents consuming alcohol depending on the frequency was statistically significant (*p* < 0.001) and showed that daily alcohol consumption was declared by more respondents in the group not participating in classes at UTA's.

Table 5 presents the characteristics of the study group, including rregular preventive examinations according to the participation in UTA's classes and Table 6 presents the characteristics of the performance of preventive tests recommended for women according to the participation in the UTA's.

**Table 5:** Regular preventive examinations according to the participation in Third Age Universities classes (*n* = 631).

**Variables**	**UTA “**+**” (*****n*** = **354)**		**UTA “–” (*****n*** = **277)**		***P*-value**
	***n***	**%**	***n***	**%**	
**Regular controls by family doctor**					
Yes	314	88.70	241	87.00	0.52
No	40	11.30	36	13.00	
**Regular annual dental controls**					
Yes	265	74,86	161	58,12	< 0.001
No	89	25,14	116	41,88	
**Regular self-examination of breasts/testicles**					
Yes	218	61.58	148	53.43	0.04
No	136	38.42	129	46.57	
**Regular laboratory tests controls**					
Yes	318	89.83	232	83.75	0.02
No	36	10.17	45	16.25	

Data are presented as absolute and relative frequencies, UTA “+”—seniors participating in the activities of the Universities of the Third Age, UTA “–”—seniors refusing to participate in the activities of the Universities of the Third Age, p-value—statistical significance.

**Table 6:** Performance of preventive tests recommended for women according to the participation in the University of the Third Age (*n* = 475).

**Variables**	**UTA “**+**” (*****n*** = **304)**		**UTA “–” (*****n*** = **171)**		***P*-value**
	***n***	**%**	***n***	**%**	
**Regular gynecological control**					
Yes	191	62.83	93	54.39	0.07
No	113	37.17	78	45.61	
**Regular cytological tests**					
Yes	178	58.55	89	52.05	0.17
No	126	41.45	82	47.95	
**Regular mammograms**					
Yes	217	71.38	108	59.65	0.06
No	87	28.62	63	36.84	

Data are presented as absolute and relative frequencies, UTA “+”—seniors participating in the activities of the Universities of the Third Age, UTA “–”—seniors refusing to participate in the activities of the Universities of the Third Age, p-value—statistical significance.

Over 85% of seniors participating and not participating in UTA's activities were under the constant care of a family doctor, while regular dental check-ups were not carried out by approximately $\frac{1}{4}$ (89; 14%) and over 40% (116; 41.88%) of them, respectively. The Chi^2^ test showed statistically significant differences in the number of seniors who regularly check their teeth (*p* < 0.001), perform self-examination of their breasts/testicles (*p* = 0.04) and perform regular laboratory tests (*p* = 0.02) over respondents attending and not attending UTA's classes.

Regular gynecological check-ups were declared by over 60% (191; 62.83%) of women participating in UTA's and approximately 55% (93; 54.39%) of those who denied the above-mentioned activity, and regular cytological tests were declared by approximately 60% (178; 58.55%) women attending and just over 50% (89; 52.05%) of those not attending UTA's. The observed differences in the numbers of individual groups were not statistically significant (*p* = 0.07 and *p* = 0.17).

Tables 7, 8 present the characteristics of the study group, including the procedure of logistic and multiple regression analyzes of the impact of sex, age, place of residence and participation in UTA's on studied health-promoting activities.

**Table 7:** Characteristic of study group taking into account logistic regression results.

**Variables**	**β**	**SE**	**Wald's χ^2^**	***p***	**OR (95% CI)**
**Spending free time**					
Sex	−0.492	0.204	5.801	0.016	0.611 (0.409–0.913)
Age	0.055	0.015	14.353	< 0.001	1.057 (1.027–1.088)
Place of residence	−0.429	0.240	3.189	0.074	0.651 (0.406–1.0437)
Attendance for UTA's classes	1.382	0.229	36.297	< 0.001	3.982 (2.538–6.248)
Model χ^2^ = 76.556; *p* < 0.001. Model fit measure (−2log): for this model = 765.437, constant term = 841.993.					
**Regular physical activity**					
Sex	−0.076	0.208	0.134	0.714	0.926 (0.615–1.395)
Age	0.063	0.015	18.520	< 0.001	1.066 (1.035–1.097)
Place of residence	0.050	0.234	0.045	0.832	1.051 (0.664–1.665)
Attendance for UTA's classes	1.205	0.228	28.058	< 0.001	3.337 (2.135–5.217)
Model χ^2^ = 73.192; *p* < 0.001. Model fit measure (−2log): for this model = 751.486, constant term = 824.678.					
**Self-assessment of physical activity**					
Sex	0.130	0.212	0.380	0.538	1.139 (0.752–1.726)
Age	0.023	0.014	2.508	0.113	1.023 (0.995–1.053)
Place of residence	−0.597	0.241	6.114	0.013	0.551 (0.343–0.884)
Attendance for UTA's classes	0.867	0.226	14.759	< 0.01	2.380 (1.528–3.706)
Model χ^2^ = 17.432, *p* = 0.002. Model fit measure (−2log): for this model = 768.454, constant term = 785.887.					
**Tobacco smoking in the past**					
Sex	−0.679	0.209	10.597	0.001	0.507 (0.337–0.764)
Age	−0.033	0.015	4.823	0.028	0.968 (0.940–0.997)
Place of residence	−0.483	0.253	3.633	0.057	0.617 (0.375–1.015)
Attendance for UTA's classes	−0.339	0.231	2.149	0.143	0.713 (0.453–1.122)
Model χ^2^ = 24.409; *p* < 0.001. Model fit measure (-2log): for this model = 774.798, constant term = 799.207.					
**Alcohol consumption**					
Sex	−0.757	0.212	12.767	< 0.001	0.469 (0.309–0.711)
Age	−0.034	0.014	5.999	0.014	0.966 (0.940–0.993)
Place of residence	0.089	0.233	0.148	0.701	1.094 (0.693–1.726)
Attendance for UTA's classes	−0.789	0.221	12.755	< 0.001	0.454 (0.295–0.701)
Model χ^2^ = 30.660; *p* < 0.001. Model fit measure (−2log): for this model = 816.517, constant term = 847.177.					
**Regular annual dental controls**					
Sex	−0.261	0.212	1.501	0.221	0.771 (0.507–1.170)
Age	−0.051	0.014	12.466	< 0.001	0.950 (0.923–0.978)
Place of residence	−0.051	0.236	0.047	0.828	0.950 (0.598–1.509)
Attendance for UTA's classes	−0.783	0.227	11.898	< 0.001	0.457 (0.293–0.714)
Model χ^2^ = 33.320; *p* < 0.001. Model fit measure (−2log): for this model = 761.577, constant term = 794.897.					
**Regular self-examination of breasts/testicles**					
Sex	−1.191	0.203	34.243	< 0.001	0.304 (0.204–0.453)
Age	0.067	0.015	21.402	< 0.001	1.070 (1.039–1.101)
Place of residence	0.068	0.189	0.131	0.717	1.071 (0.738–1.553)
Attendance for UTA's classes	< 0.001	0.011	< 0.001	0.994	1.000 (0.978–1.022)
Model χ^2^ = 64.266; *p* < 0.001. Model fit measure (−2log): for this model = 792.512, constant term = 856.778.					
**Regular laboratory tests controls**					
Sex	−0.846	0.262	10.416	0.001	0.429 (0.257–0.718)
Age	−0.007	0.020	0.120	0.729	0.993 (0.954–1.034)
Place of residence	0.604	0.333	3.296	0.069	1.829 (0.952–3.514)
Attendance for UTA's classes	−0.082	0.338	0.059	0.808	0.921 (0.475–1.788)
Model χ^2^ = 20.183; *p* < 0.001. Model fit measure (−2log): for this model = 463.231, constant term = 483.414.					

**Table 8:** Characteristic of study group taking into account multiple regression results of time spent on physical activity.

**Duration of physical activity**				
**Variables**	β	**SE**	**t-statistics**	***p***
Coefficient	149.757	19.173	7.811	< 0.001
Sex	−8.188	3.690	−2.219	0.027
Age	−1.213	0.256	−4.739	< 0.001
Place of residence	−8.010	4,328	−1.851	0,064
Attendance for UTA's classes	−11.104	4.045	−2.745	< 0.01

R = 0.277.

R-squared = 0.077.

Adjusted R-squared = 0.071.

F = 12.929.

P ≤ 0.001.

Estimation error = 38.078.

All logistic regression models created for individual health-promoting behaviors in order to determine the impact of gender, age, place of residence and attending classes at UTAs on the likelihood of engaging in health-promoting behaviors vs. not engaging in them turned out to be statistically significant. Only models describing the impact of additional factors on tobacco smoking in the past (*p* = 0.143), regular laboratory tests (*p* = 0.808) and regular self-examination of breast/testicles (*p* = 0.994) showed no impact of attending UTA's classes.

We tested if sex, age, place of residence and attendance for UTA's classes significantly predicted participants' time spent on physical activity. The results of the regression indicated the three predictors explained 7.7% of the variance (R^2^ = 0.077, F = 12.292, *p* < 0.001). It was found that sex (β_1_ = −8.189, *p* < 0.027), age (β_2_ = −1.213, *p* < 0.001) and attendance for UTA's classes/lectures significantly predicted longer duration of physical activity (β_3_ = −11.104, *p* < 0.01).

## Discussion

Conducted study proved positive impact of UTA's attendance at examined healthy behaviors.

Seniors attending UTA's classes were more active considering all physical activity indicators, probably because they had the opportunity to participate in physical activities organized by the UTA's to which they belonged, and also because thanks to the knowledge acquired during classes, they managed their free time more effectively (15). Similar results were obtained in our other study conducted in a group of seniors living in the cities of the Silesian agglomeration (20). Regular physical activity affects, among other things: increasing the efficiency of the circulatory system, lowering blood pressure, increasing the stroke volume of the heart and improving the elasticity of blood vessels and reducing the risk of developing atherosclerosis and its complications, reducing the risk of stroke, improving metabolism, and consequently therefore, treatment of obesity and overweight, reduction of stress, improvement of cognitive functions and improvement of logical thinking processes, as well as concentration of attention and memory (21–24). Research shows that regular physical activity promotes a better quality of life and even its extension (22, 25).

Our study also examined the use of stimulants by seniors. Less than 10% of the respondents declared that they smoked tobacco. However, Bartoszek et al. showed that almost 30% of the seniors they surveyed had contact with tobacco (26). As can be read in the 2019 Research Communication of the Public Opinion Research Center “Cigarette smoking”, one fourth of adult Poles (26%) smoked tobacco. Among them, regular smokers constituted 82%, and occasional smokers-−18%, and the quoted result has not changed since 2012, similar research was conducted (27). However, according to the Report from a nationwide survey on attitudes toward smoking, prepared by Kantar for the Chief Sanitary Inspectorate—(also in 2019), over one fifth of Poles (21%) admitted to smoking tobacco every day (28). Tobacco smoking is a risk factor for the development of cancer and is also a classic cardiovascular risk factor (29). There are many reports in the literature regarding the prevalence of smoking among young and middle-aged people, but not among seniors (30–32). Observed lack of differences both in passive and active smoking could be due to: small number of study group participants who smoked (giving up the addiction in the past -significant differences in respondents numbers) or were exposed to passive smoking. Moreover, age-related limitations, such as: increased time spent at home and reduced time spent in the company of smokers or lack of funds to buy tobacco could have played role in shaping of the results (33).

Daily alcohol consumption was declared by more seniors among the participants who did not take part in the activities of the UTA's. Observed results are disturbing and might be caused by various factors. One of them can be worst knowledge about the dangers of drinking alcohol. The study by Bartoszek et al. cited above showed that as many as 83.6% of seniors drank alcohol, although they indicated that they drank alcohol occasionally (26). Mihailovic et al. showed that the prevalence of alcohol consumption among people over 55 years of age in Serbia and Hungary was 41.5% and 62.5%, respectively. In both countries, alcohol was consumed more often by men than women (34). According to analyzes by the Organization for Economic Co-operation and Development (OECD), one Pole consumes on average 11.7 liters of pure alcohol per year. Taking into account gender, it should be noted that men in our country consumed 18.4 liters of pure alcohol per year, and women−5.6 liters (35). According to WHO, the average consumption of pure alcohol in Poland exceeds the European average. Alcohol abuse not only leads to the development of many cardiovascular diseases, cancer, gastrointestinal and endocrine problems and many others, but also leads to severe addictions, injuries and aggression. Mortality caused by alcohol consumption is higher than by diseases such as tuberculosis or AIDS (36).

As indicated by the results of the European Health Interview Survey (EHIS), older people often undergo basic preventive examinations (37). More than 85% of all surveyed seniors were under the constant care of a family doctor, while seniors participating in UTA's classes were more regular in performing breast/testicular self-examination, regular laboratory tests, and checking their teeth, compared to seniors not participating in the above-mentioned classes. Regular performance of the above-mentioned health-promoting activities and the observed differences between UTA “+” and UTA “–” seniors may result from the information presented during the classes and the knowledge acquired during them. Therefore, it can be concluded that UTA's students had better knowledge of health prevention and the benefits of regular examinations. Similar results were obtained in our previously cited other studies conducted in a group of seniors. However, there it was mainly due to living in cities and better access to medical services (20).

Regular gynecological check-ups were declared by over 60% of women participating in UTA's activities. Preventing reproductive system diseases in women requires regular check-ups with a doctor. In the study conducted in a group of younger women than in our study (age over 40), on average every third woman (41; 36%) had regular check-ups. Respondents reported for cytological examination at similar intervals as for check-up visits to a gynecologist—most often once a year (44; 39%) (38). Similar data were also obtained by Bojar et al. in a study on a group of 304 women, most of whom underwent cytology once a year (39). In Stanisławska's research, as many as 93.9% of respondents declared that they performed gynecological examinations once a year (40). In Poland, the cervical cancer prevention program, which includes cytological examination every 3 years, is addressed to women aged 25 to 64. After this time, you can consider stopping regular testing, but only if the results are in normal ranges.

Our study did not show a difference in the number of women regularly undergoing mammography examinations depending on their attendance at UTA's classes. Observed result can be caused by women age or lack in the knowledge of the importance of mammography. Pivot et al., in a group of French women aged 40–74, showed that the surveyed women underwent regular examinations. Similar results can be found in the Report of the Central Statistical Office (GUS), however, both of these studies were conducted in groups of younger women than our own studies (41, 42). Mammography is a recognized diagnostic method for the early detection of breast cancer. Population-based breast cancer prevention programs are aimed at women over 50 years of age. Similarly to cytology, in Poland the breast cancer prevention program, which includes mammography every 2 years, is addressed to women aged 45 to 74. After this time, you can consider quitting the regular test, but only if the results were correct, which may also affect the results obtained in your own study.

According to many studies and reports, Poles' eating habits are bad. The National Institute of Public Health of the National Institute of Hygiene (NIZP-PZH) in the study entitled: “The health situation of the Polish population and its determinants in 2020” revealed that: in the years 2010–2018, the amount of bread and flour eaten among Poles decreased by over 30%, the consumption of potatoes decreased by 35%, other vegetables—by 6%, and fruit consumption—increased by 6%. During the mentioned period, Poles increased their consumption of red meat products by as much as 120%. As it also turned out, an average Pole ate from 120 to 129 g of red meat and various meat products per day, meanwhile, according to the World Cancer Research Found (WCRF) and the American Institute for Cancer Research (AICR), the consumption of red meat for an adult should not exceed 71 grams. Fish consumption also decreased by as much as 40%. However, the number of confectionery products eaten by Poles, as well as crispbread, rice wafers and other bakery products, increased by 20% (43, 44). Our study showed that the analyzed groups of seniors did not differ significantly from each other in terms of the points obtained in the My Eating Habits questionnaire. The results of the current study indicated that participating in UTA activities might not influence eating habits. The lack of differences in eating habits could be caused by the occurrence of selected diseases for which specialized diet is an element of treatment and tertiary prevention, both among UTA's”+” and UTA “–” respondents. Compliance with the diet by at least some of the respondents could have influenced the obtained results.

In the regression models we found out a statistically significant impact of the factors included in the models (sex, age, place residence, attendance for UTA's classes) on health-promoting behaviors under study. Presented regression models showed that attendance in UTA's classes influenced taking health-promoting activities in the discussed areas.

Limitations of the study include but are not limited to: relatively small sample size, self-reported data by the participants, possible occurrence of Hawthorne's effect when obtaining answers and examined scope limited only to few health behaviors and possibly influencing them variables. Another limitation of the study is the fact that the authors did not analyze the exact content of the classes/lectures in which the surveyed seniors participated. This was due to restrictions on third parties wanting to take part in classes/lectures and the copyright of speaker's classes/lectures on a given topic. However, it was checked whether in a given academic year, classes on all the discussed health-promoting activities were held at a given University of Third Age.

Taking into account obtained results, UTA's future activities should focus on further education and making seniors and the constantly aging society aware of the benefits of adopting health-promoting behaviors, with particular emphasis on their impact on the quality and length of life.

Further research conducted in this area, on larger groups of respondents, should focus on further exploration of health-promoting behaviors undertaken in various groups of seniors, taking into account additional factors, such as existing diseases. The results obtained on the basis of this and further research regarding the positive effect of UTA's on the lives of seniors may support people managing various educational and local governmental institutions to take actions related to managing an increasing number of UTA's not only in Poland but also in other countries.

## Conclusions

Health-promoting behaviors of seniors attending classes at the UTA's were more correct in terms of physical activity, adequate attendance with preventive test and worst in terms of alcohol consumption. Overall picture allows to conclude that participation in UTA's classes seems to have a positive impact on the examined health-promoting behaviors of the surveyed seniors. Nevertheless, there is a need to conduct educational activities to promote the benefits of participating in the activities of UTA's, contributing to an active and health-promoting lifestyle and, consequently, extending and improving its quality.

## Data availability statement

The raw data supporting the conclusions of this article will be made available by the authors, without undue reservation.

## Ethics statement

The studies involving humans were approved by Bioethics Committee of the Medical University of Silesia in Katowice (PCN/0022/KB1/36/21). The studies were conducted in accordance with the local legislation and institutional requirements. The participants provided their written informed consent to participate in this study.

## Author contributions

JD: Conceptualization, Formal analysis, Investigation, Methodology, Project administration, Software, Supervision, Validation, Visualization, Writing – original draft, Writing – review & editing. MS: Data curation, Formal analysis, Investigation, Methodology, Resources, Validation, Writing – original draft, Writing – review & editing. OS: Formal analysis, Resources, Writing – original draft, Writing – review & editing. EŁ: Data curation, Methodology, Writing – original draft. HK: Data curation, Resources, Software, Writing – original draft.
